# Sensory deficit screen identifies *nsf* mutation that differentially affects SNARE recycling and quality control

**DOI:** 10.1016/j.celrep.2023.112345

**Published:** 2023-04-05

**Authors:** Yan Gao, Yousuf A. Khan, Weike Mo, K. Ian White, Matthew Perkins, Richard A. Pfuetzner, Josef G. Trapani, Axel T. Brunger, Teresa Nicolson

**Affiliations:** 1 Department of Otolaryngology, Head and Neck Surgery, Stanford Medical School, 300 Pasteur Drive, Stanford, CA 94303, USA; 2 Department of Molecular and Cellular Physiology, Stanford University, Stanford, CA, USA; 3 Department of Neurology and Neurological Sciences, Stanford University, Stanford, CA, USA; 4 Department of Structural Biology, Stanford University, Stanford, CA, USA; 5 Department of Photon Science, Stanford University, Stanford, CA, USA; 6 Center for Biomedical Informatics Research, Stanford University, Stanford, CA, USA; 7 Graduate Program Biomedical Sciences, Oregon Hearing Research Center and Vollum Institute, Oregon Health and Science University, 3181 SW Sam Jackson Park Road, Portland, OR 97239, USA; 8 Howard Hughes Medical Institute, Stanford University, Stanford, CA, USA; 9 Department of Biology and Neuroscience Program, Amherst College, Amherst, MA 01002, USA; 10 These authors contributed equally; 11 Lead contact

## Abstract

The AAA^+^ NSF complex is responsible for SNARE complex disassembly both before and after membrane fusion. Loss of NSF function results in pronounced developmental and degenerative defects. In a genetic screen for sensory deficits in zebrafish, we identified a mutation in *nsf*, I209N, that impairs hearing and balance in a dosage-dependent manner without accompanying defects in motility, myelination, and innervation. *In vitro* experiments demonstrate that while the I209N NSF protein recognizes SNARE complexes, the effects on disassembly are dependent upon the type of SNARE complex and I209N concentration. Higher levels of I209N protein produce a modest decrease in binary (syntaxin-SNAP-25) SNARE complex disassembly and residual ternary (syntaxin-1A-SNAP-25-synaptobrevin-2) disassembly, whereas at lower concentrations binary disassembly activity is strongly reduced and ternary disassembly activity is absent. Our study suggests that the differential effect on disassembly of SNARE complexes leads to selective effects on NSF-mediated membrane trafficking and auditory/vestibular function.

## In brief

Gao et al. combine forward genetics with biochemical analysis to provide mechanistic insight into a sensory deficit. Both *in vivo* and *in vitro* data highlight the differential effect of the I209N mutation on development and SNARE disassembly, indicating a key role for quality control by NSF in a biological context.

## Graphical Abstract

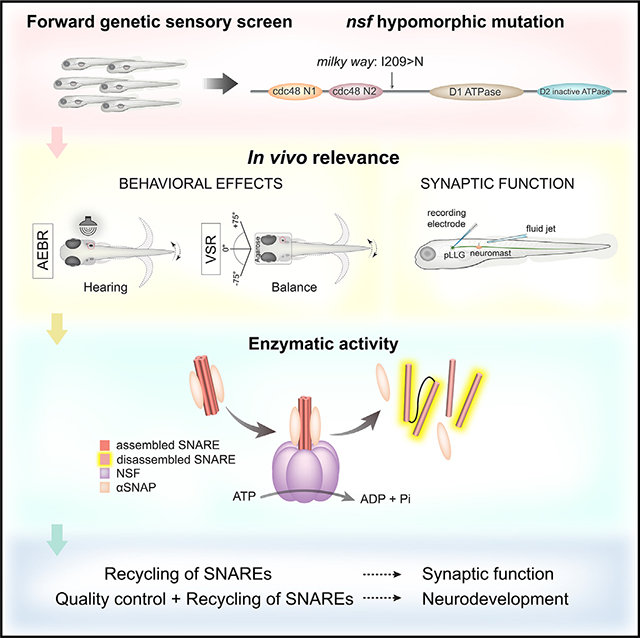

## INTRODUCTION

Vesicle fusion is vital to both intracellular trafficking of proteins and release of extracellular proteins and neurotransmitters. Membrane-bound or -embedded SNARE proteins on opposing membrane compartments are essential for mediating vesicle fusion in multiple cellular contexts. For example, in neurons the SNARE proteins syntaxin-1, SNAP-25, and synaptobrevin-2 are primarily localized on separate membranes—the plasma membrane and the synaptic vesicle membrane, respectively. These SNARE proteins zipper together to form a highly stable coiled-coil *trans* ternary complex, bringing opposing membranes close together and providing the requisite energy for membrane fusion of synaptic vesicles.^1,2^ After fusion, the fully formed *cis* SNARE complex resides in the plasma membrane. Prior to engagement in the trans SNARE complex, syntaxin-1 and SNAP-25 can form a binary complex. Although it can act as an acceptor complex for synaptobrevin-2, the binary complex forms a variety of conformations and stoichiometries, some of which are kinetic dead-ends that do not lead to fusogenic *trans* SNARE complex. Both postfusion ternary SNARE *cis* complexes and prefusion binary SNARE complexes are disassembled by *N*-ethylmaleimide-sensitive factor (NSF) together with SNAP. Thus, NSF/SNAP are part of a recycling and quality control system for SNAREs.^3,4^ NSF is an AAA^+^ ATPase that forms a ring-shaped homohexamer that couples ATP hydrolysis to the mechanical disassembly of SNARE protein complexes. NSF is composed of three domains, an N domain, the first AAA^+^ domain (D1), and the second AAA^+^ domain (D2). The D2 domain is primarily responsible for oligomerization, while the D1 domain actively processes substrate over successive rounds of hydrolysis. The N domain interacts with the SNAP adaptor proteins. Structures of full-length NSF and complexes with SNAPs and SNAREs have been determined using cryoelectron microscopy (cryo-EM).^5,6^

NSF is ubiquitously expressed in eukaryotes, and deletions in many species lead to deleterious effects. Complete loss of NSF results in pronounced developmental or degenerative defects, ultimately leading to lethality. Knockout of *NSF* is larval or embryonically lethal in flies and mice.^7,8^ In humans, *de novo* mutations in *NSF* have been associated with infantile epilepsy, severe neurodevelopmental defects, and early death.^9^ In zebrafish, the *nsf* gene is duplicated. Knockdown of both genes reduces embryonic viability.^10^ In single *nsf* zebrafish mutants, lethality occurs at larval stages, likely due to maternal transcripts that permit early development to proceed normally.^11^ Despite partially over-lapping expression patterns, mutations in *nsfa* or *nsfb* result in different phenotypes. The *nsfb* ohnolog is more broadly expressed, and loss of *nsfb* function causes hypopigmentation along with early degeneration and necrosis at 3–4 days post fertilization (dpf).^12^ Both the system-wide function of *nsfb* and early lethality in *nsfb* nulls suggest that *nsfb* is critical for intracellular trafficking and exocytosis in many cell types during the embryo-to-larva transition. Unlike *nsfb*, expression of *nsfa* is mainly restricted to the nervous system in zebrafish, and null mutants survive beyond 5 dpf.^10,11,13^ Nevertheless, previous studies suggest that null mutations in *nsfa* also affect membrane trafficking, leading to abnormal development or degenerative effects in neurons, glial cells, and sensory systems. Pronounced phenotypes in *nsfa* mutants include paralysis, expanded melanophore pigment cells due to visual defects, a severe reduction in the myelination of axons, and deinnervation of sensory hair cells.^11,13^ Why myelination in *nsfa* mutants is defective is not clear; however, plasma membrane production, which relies on exocytosis, is essential for myelination.^14^ Defective exocytosis is also thought to cause deinnervation of hair cells due to the absence of secreted neurotrophic factors such as Bdnf.^13^ In addition, secretory defects in the larval hypothalamus have also been described for *nsfa* mutants.^10^ Presumably in response to the loss of exocytosis, *nsfb* transcripts are upregulated in *nsfa* null mutants.^10,13^ Nevertheless, upregulation of *nsfb* does not compensate for the *nsfa* phenotype. The evolutionary split in expression and function of the zebrafish *nsf* genes is a common feature of duplicated genes,^15^ and this aspect of the zebrafish model system can be advantageous for more in-depth analyses of protein function. Collectively, the aforementioned *in vivo* studies of NSF highlight the importance of constitutive membrane fusion in development and survival across species.

In this study we report the identification of a unique zebrafish auditory/vestibular mutant from a random mutagenesis screen with a missense mutation in *nsfa*. The allele is predicted to result in an amino acid substitution of asparagine (N) for isoleucine (I) at position 209. In contrast to *nsfa* null mutations,^13^ the morphology of hair-cell synapses is not disrupted by the I209N mutation, indicating that the I209N allele is hypomorphic. Recordings of evoked and spontaneous action potentials from hair-cell afferent neurons reveal that Nsfa is required for both accurate temporal transmission of mechanical stimuli at hair-cell synapses and recovery of spontaneous vesicle release after sustained stimulation. *In vitro* experiments demonstrate that the I209N mutant NSF protein together with αSNAP form complexes with either binary or ternary SNARE complexes; however, the disassembly of these SNARE complexes is differentially affected by the I209N mutation. At the higher concentration of NSF that was tested here, the I209N mutation mildly reduces binary complex disassembly and greatly reduces ternary complex disassembly. At a lower concentration there is a more pronounced effect on binary complex disassembly and no detectible ternary complex disassembly. This differential effect on activity, which occurs in an NSF-concentration-dependent fashion, suggests that the I209N mutation is more detrimental to the postfusion disassembly activity of NSF in contrast to its prefusion activity. Taken together, these findings are consistent with the hypomorphic nature of the I209N allele and further illustrate the critical role of NSF in synaptic function.

## RESULTS

### An *nsfa* point mutation identified in *milky way* mutants

**Figure 1.**
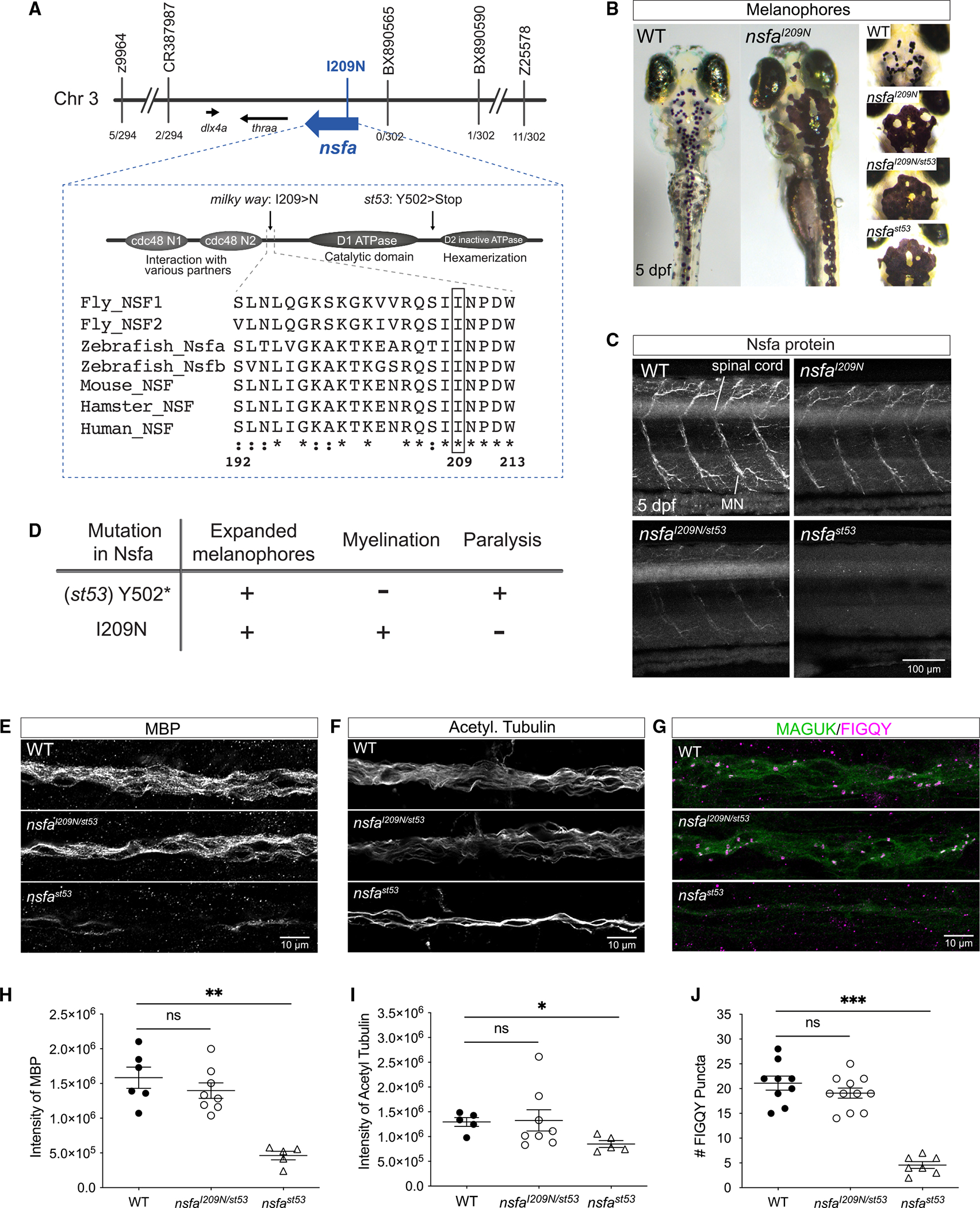
Identification of *milky way* as a hypomorphic allele of *nsfa* (A) Map of the critical region. A missense mutation was identified in *nsfa*. The null allele of *nsfa* (*st53*) is also indicated. Protein sequence of the linker region of NSF near I209 (boxed) from various species. (B)Expanded melanophore phenotype. (C)Nsfa immunolabeling in spinal cord and motor neurons in WT, *nsfa*^*I209N*^ homozygous mutants, d*nsfa*^I*209N*/*st53*^ compound heterozygotes, and *nsfa*^*st53*^ homozygous mutants. Scale bar, 100 μm. (D)Summary of phenotypes in the null and I209N *nsfa* mutants. (E–J) Myelination and the number of nodes of Ranvier in the lateral-line nerve of mutant *nsfa*^I*209N*/*st53*^ larvae are comparable with that seen in WT siblings. Representative images (E–G) and quantification (H–J) of MBP, AcTub, and FIGQY antibody labeling (with colabeling of the postsynaptic density by MAGUK) in WT, *nsfa*^*I209N*/*st53*^, and *nsfa*^*st53*^ larvae. Images are maximum intensity z projections from the initial segment of the posterior lateral-line nerve. Quantification data are shown as mean ± SEM; p values were determined by Student’s t tests to compare with WT group. ns, not significant; *p < 0.05, **p < 0.01, ***p < 0.001. For all experiments, n ≥ 5 fish per genotype (5 dpf). All images and data are representative of 2 or 3 independent experiments. Scale bar, 10 μm.

The fully penetrant *milky way* mutation was isolated in a large-scale screen for zebrafish larvae with hearing or balance defects (Tubingen 2000 screen). Homozygous *milky way* mutants exhibit an escape response to loud acoustic stimuli such as vigorous tapping on the Petri dish, yet the larvae display obvious balance defects while swimming. The uncoordinated motility in these mutants is lethal, with death occurring when the yolk nutrients are exhausted (~10 dpf). Meiotic mapping using simple sequence length polymorphism markers on 302 mutants from eight pairs of heterozygous fish located the mutation to a 400-kb critical region (Figure 1A). Sequencing of cDNA from three genes in the region identified a point mutation in *nsfa*, an ohnolog of *nsf*. The recessive T>A point mutation in *milky way* mutants results in a change from an isoleucine to an asparagine residue within the linker region between major domains in the protein (I209N, *nsfa*^*I209N*^, Figure 1A). This isoleucine residue is conserved across eukaryotic species from yeast to human, indicating its importance to NSF function (Figure 1A).

To confirm the identity of the gene, we performed complementation crosses with heterozygous fish carrying a nonsense mutation in *nsfa*, *st53* (Figure 1A), which has been previously shown to be a null allele.^13,16^ Like homozygous *nsfa*^*st53*^ mutants, homozygous I209N mutant larvae have expanded dark pigment cells known as melanophores (Figure 1B). Expanded melanophores in larvae are indicative of a visual impairment.^11,17^ The resulting compound mutant progeny carrying one copy of *I209N* allele combined with the null allele (*I209N*/*st53*) also had expanded melanophores (Figures 1B and S1B). Importantly, the auditory/vestibular defects were still present in the compound mutants, indicating that *nsfa* is the causative gene.

### Protein and transcript levels in *nsfa* mutants

To determine the effects of the missense mutation on Nsfa protein levels, we immunolabeled larvae with antibodies against human NSF. In Figure 1C, the level of Nsfa protein in the spinal cord and motor neurons is shown in wild-type (WT) siblings, homozygous *I209N*, compound *I209N*/*st53*, and homozygous *st53* mutants. Immunolabeling was largely restricted to the nervous system, which is consistent with previous reports.^11,13,18^ The levels of Nsfa in the nervous system were lower in the *I209N* homozygous mutants compared with WT siblings, and Nsfa protein was further reduced in compound mutants and absent in the null mutant (*st53*). Owing to the auditory and vestibular deficits present in the *nsfa*^*I209N*^ mutant, we focused on the acousticolateralis system and quantified the levels of Nsfa in sensory hair cells of the lateral-line organ (Figure S2A). As seen in the other types of neurons (Figure 1C), Nsfa levels in this cell type were significantly reduced in either homozygous or compound I209N mutants in comparison with WT Nsfa levels (Figures S2A and S2B). Higher levels of Nsfa protein were detected in the hair cells of I209N homozygous mutants, reflecting the copy number of the I209N allele.

Although Nsfa protein levels were reduced in I209N mutants, we found that transcripts for *nsfa* were slightly elevated using quantitative PCR (Figure S2C). This effect was specific to the I209N allele and not observed in fish carrying the *st53* null allele. To test for potential compensation in I209N mutants by *nsfb*, we also examined transcript levels for this gene duplicate and observed that *nsfb* transcripts were not significantly changed in the *nsfa*^*I209N*^ or *nsfa*^*I209N*/*st53*^ mutants (Figure S2C). In contrast, the *st53* null allele led to nonsense-mediated decay of *nsfa* transcripts, and *nsfb* was upregulated instead (Figure S2C). Our results are consistent with a previous report by Kurrasch et al.^10^ showing that *nsfb*, but not *nsfa*, is upregulated in *nsfa*^*st53*^ mutants. This type of compensatory response is common to nonsense mutations if a gene duplicate is present.^19^ However, in this case upregulation of *nsfb* transcripts was not sufficient to compensate for the *st53* phenotype of paralysis and reduced myelination, highlighting their nonoverlapping roles. Together, these results suggest that the I209N protein is expressed in neuronal cell types and that transcriptional regulation of the closely related ohnolog *nsfb* is unchanged in the I209N mutant.

### Lack of myelination and paralysis defects in I209N mutants

Previous studies of *nsfa*^*st53*^ zebrafish have shown that Nsfa is also essential for myelination of axons of the lateral-line system.^11,13^ The myelin sheath and clustering of voltage-gated sodium channels at the nodes of Ranvier are well-known requirements for fast propagation of action potentials in vertebrates. Defects in myelination and node formation, particularly in acousticolateralis afferent neurons, could therefore potentially explain the behavioral defects in the *I209N* mutants.

To assess myelin sheaths and the nodes of Ranvier, we examined several components of these axonal structures in the lateral-line system of *nsfa*^*I209N/st53*^ larvae, which are predicted to have a more severe phenotype. We used antibodies against myelin basic protein (MBP) and acetylated tubulin (AcTub) to label the myelin sheaths and axons of the lateral-line nerve, respectively. Like other species, MBP is an abundant protein in the myelin sheath,^20^ and other studies have also used MBP expression to assess myelination of lateral-line neurons in zebrafish larvae.^13,21,22^ As expected, the expression levels and patterns of both MBP and AcTub were dramatically changed in 5-dpf *nsfa*^*st53*^ mutants (Figures 1E, 1F, 1H, and 1I). In contrast, the labeling of MBP and AcTub in *nsfa*^*I209N/st53*^ mutants was similar to that seen in WT larvae. Although it has been shown that the *nsfa*^*st53*^ mutation in zebrafish affects both myelination and nodes of Ranvier,^11^ mutations in other zebrafish genes have been found to disrupt the mature node of Ranvier without disrupting the formation of myelin sheath.^23^ To test for a more selective defect on myelin organization, we used an FIGQY antibody, which recognizes conserved peptides present at nodes of Ranvier.^11,24^ In comparison with *nsfa*^*st53*^ mutants that have drastically reduced clustering of FIGQY labeling, both WT and *nsfa*^*I209N/st53*^ larvae showed comparable numbers of FIGQY clusters (Figures 1G and 1J). Our results indicate that the *nsfa*^*I209N*^ point mutation in zebrafish does not affect myelination and clustering of the nodes of Ranvier in lateral-line neurons.

**Figure 2.**
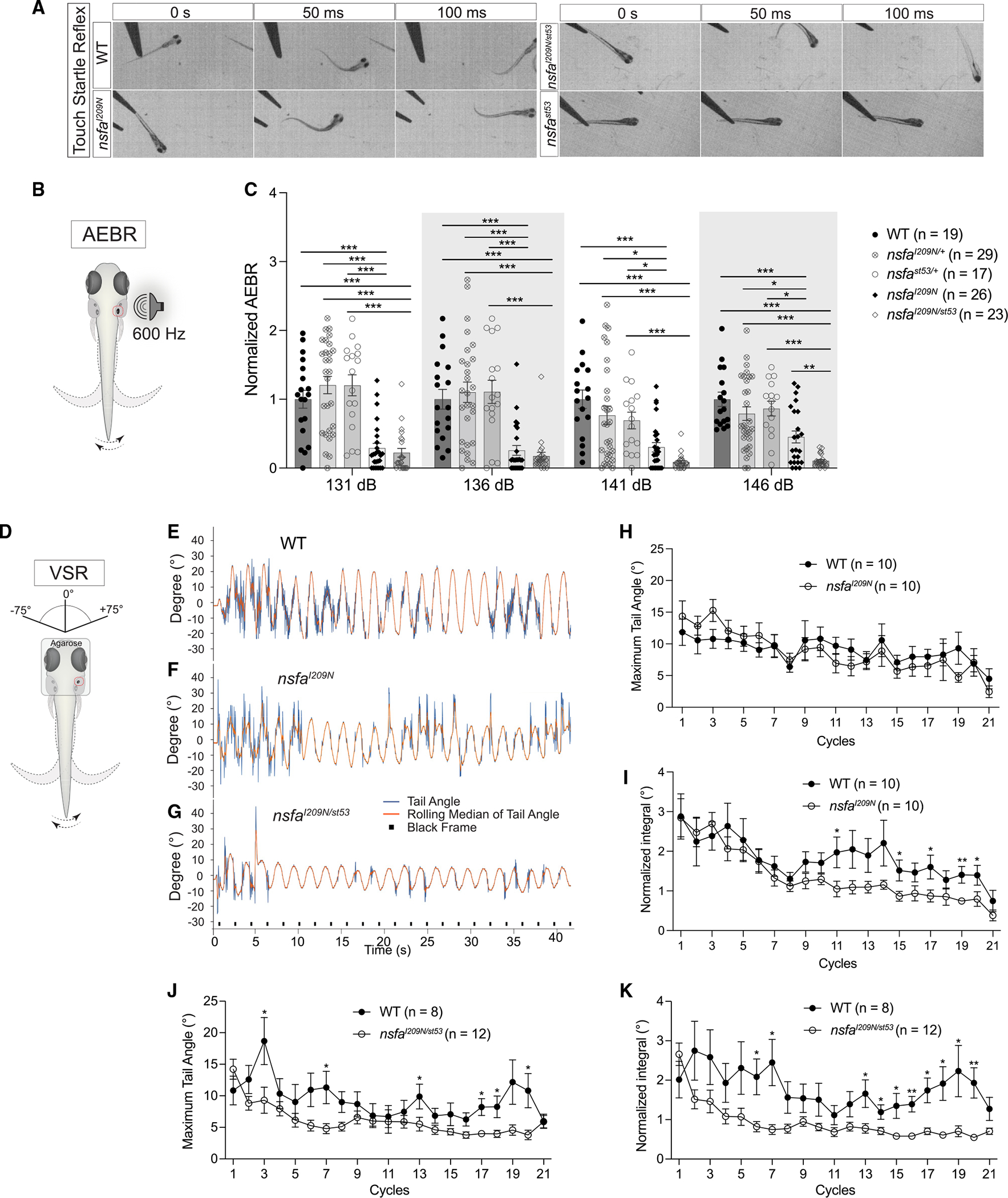
The I209N mutation in Nsfa selectively affects hearing and vestibular function without causing paralysis (A) Still frames from high-speed videos of larvae. *nsfa*^*st53*^ homozygotes were paralyzed and unresponsive to touch, whereas *nsfa*^*I209N*^ homozygotic and *nsfa*^*I209N*/*st53*^ larvae displayed robust startle reflexes. (B) (B and C) Acoustic evoked behavioral responses of larvae exposed to a 600-Hz stimulus at the intensities indicated normalized to homozygous WT siblings. n indicates number of fish tested. (D–K) Vestibulospinal reflexes are reduced in *nsfa*^*I209N*^ homozygotic and *nsfa*^*I209N*/*st53*^ larvae. (E–G) Raw traces (blue) of the tail movements of representative WT and mutant larvae. The rolling median (orange trace) is a movement artifact. (H–K) The maximum tail angle (H and I) and the normalized integral (J and K) of sibling cohorts were quantified using ZebraZoom. n indicates number of fish tested. Mean ± SEM and two-way ANOVA with Benjamini-Hochberg correction for each dataset were performed. *p < 0.05, **p < 0.01, ***p < 0.001.

As described previously, the *nsfa*^*st53*^ null allele causes severe paralysis in 5-dpf larvae.^10,11,13^ In contrast, both the homozygous and compound I209N mutants did not display a motility defect and responded normally to touch stimuli (Figure 2A). Collectively, our molecular and phenotypic analyses thus far suggested that the *nsfa*^*I209N*^ is a hypomorphic allele of *nsfa* with partial function that selectively affects the acousticolateralis system (Figure 1D).

### Hearing and balance defects in *nsfa*^*I209N*^ mutants

Similar to zebrafish mutants with more subtle hair-cell phenotypes,^25–27^ *nsfa*^*I209N*^ mutants respond to coarse acoustic stimulation but fail to consistently orient their body to gravity when they swim. To determine the extent of vestibular dysfunction and whether an auditory deficit in *nsfa*^*I209N*^ mutants is present, we utilized more sensitive behavioral assays to quantify the nature and extent of hearing and balance deficits in the mutants.

We first examined the contribution of *nsfa* to auditory function via the auditory evoked behavioral response (AEBR). The AEBR is a simple measurement of escape behavior in response to acoustic stimuli^26,28^ (Figure 2B). We assessed the AEBR using pure tones of 600 Hz at four different intensities (131–146 dB, Figure 2C). Both homozygous and compound mutants had strongly reduced acoustic startle responses, with compound mutants being less responsive than homozygous I209N mutants at 146 dB. This phenotype was striking, prompting us to test for haploinsufficiency among the heterozygous WT larvae; however, we did not observe any statistically significant differences between homozygous and heterozygous WT siblings (Figure 2C). Given the lack of morphological defects in myelination of afferent neurons, the reduction in the AEBR suggests that partial loss of hearing may be due to a disruption of hair-cell function in both mutants.

To assess vestibular function, we examined the vestibulospinal reflex (VSR), which tests motor output of the trunk in response to head rotations^29^ (Figure 2D). Representative tracking traces showed that there were fewer tail-movement responses to the rotary stimulation in both homozygous and compound mutants compared with the WT larvae (Figures 2E–2G, blue traces). To quantify the VSR, we analyzed the maximum tail angle of the movements and the normalized integral of the tail movements during each cycle of rotation. Homozygous I209N mutants did not differ in terms of maximum tail angles (Figure 2H). In contrast, *nsfa*^*I209N*/*st53*^ mutants produced significantly reduced maximum tail angles compared with WT siblings (Figure 2I). The normalized integral, which reflects the level of activity during each cycle, was decreased in both mutants, with greater effects seen in the compound mutants (Figures 2J and 2K). Together, the behavioral data indicate that both the vestibular system and, to a lesser extent, the auditory system are sensitive to the I209N mutation in a gene-dosage-dependent manner.

### Hair-cell ribbon synapses are normal in *nsfa*^*I209N/st53*^ mutants

**Figure 3.**
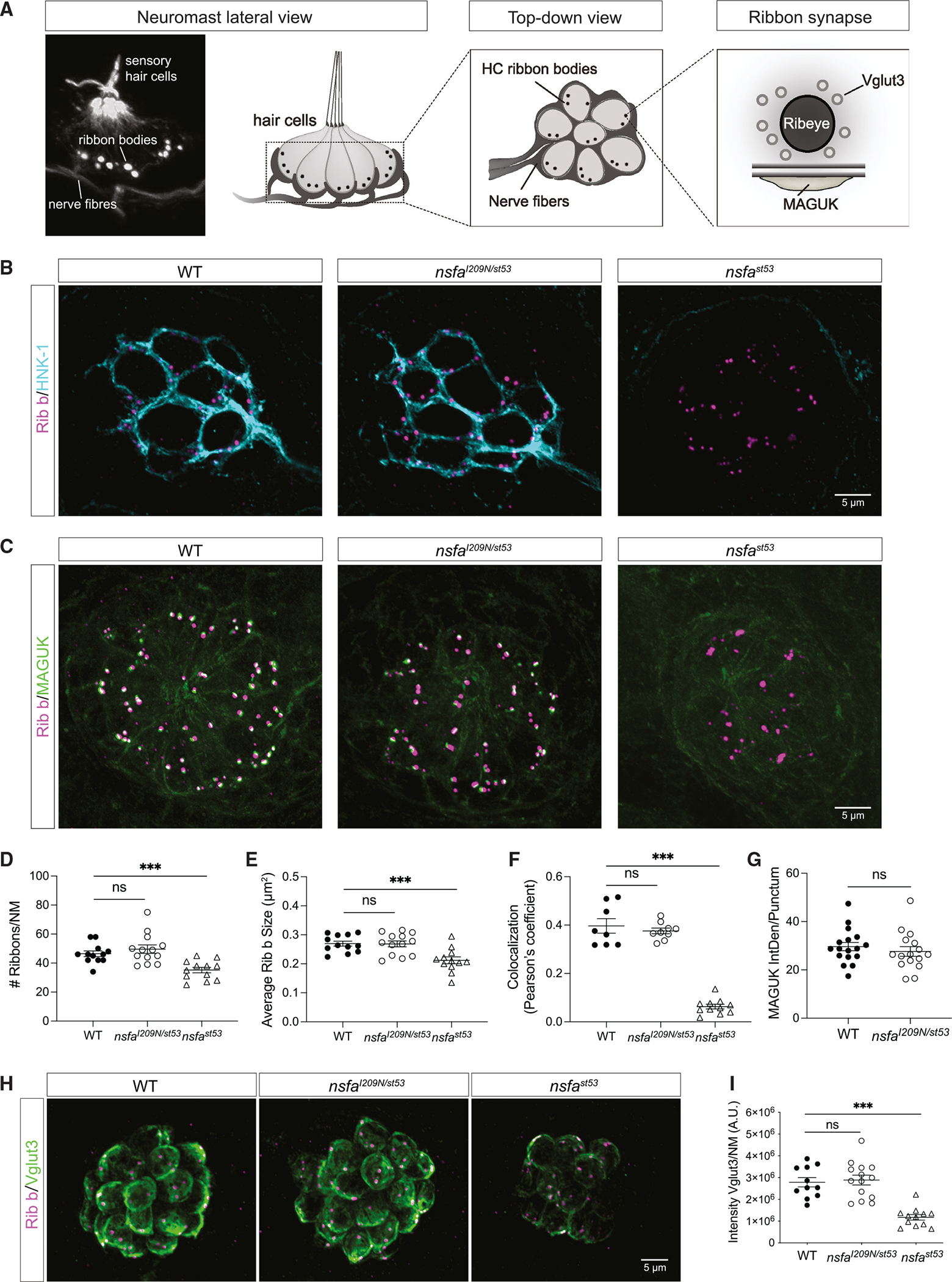
The gross morphology of ribbon synapses is normal in *nsfa*^*I209N/st53*^ mutants (A) Image and diagrams of neuromasts depicting the hair cells, afferent neurons, and ribbon synapses. (B) Representative images of afferent innervation (HNK-1) of hair cells in WT, *nsfa*^*I209N*/*st53*^ mutants, and *nsfa*^*st53*^ mutants. Presynaptic hair-cell ribbons are labeled with Ribeye b antibody (magenta). Note the lack of innervation of *nsfa* null hair cells. (C) Ribbon synapses in WT, *nsfa*^*I209N*/*st53*^ mutants, and *nsfa*^*st53*^ mutants. A pan-MAGUK antibody was used to label the postsynaptic density of afferent terminals (green), and ribbons were visualized with anti-Ribeye b antibody (magenta). (D) Number of ribbons per neuromast (n ≥ 12 neuromasts per genotype). (E) Average size of each ribbon (n ≥ 12 neuromasts per genotype). (F) Colocalization of Ribeye b and MAGUK (n ≥ 8 neuromasts per genotype). (G) Integrated density of MAGUK immunolabel per punctum (n ≥ 12 neuromasts per genotype). (H) Images of the synaptic vesicle marker VGlut3 in WT, *nsfa*^*I209N*/*st53*^ mutants, and *nsfa*^*st53*^ mutants (Ribeye b in magenta). (I) Quantification of the intensity of the VGlut3 immunolabel (n ≥ 11 neuromasts per genotype). Quantification data are shown as mean ± SEM; p values are determined by ANOVA tests to compare with WT group. ns, not significant; *p < 0.05, **p < 0.01, ***p < 0.001. For all experiments, n ≥ 6 fish per genotype (5 dpf). All images and data are representative of 2 or 3 independent experiments. Scale bars, 5 μm.

Nsfa is required for the maintenance of innervation and integrity of hair-cell synapses;^13^ therefore, we examined the morphology of ribbon synapses in the more strongly affected *nsfa*^*I209N*/*st53*^ mutants (diagrammed in Figure 3A). To visualize the neurite extensions of afferent neurons to hair cells, we labeled them with an HNK-1 antibody, which specifically labels afferent fibers, and colabeled hair cells with anti-Ribeye b antibodies that label presynaptic ribbon bodies. As seen in Figure 3B, innervation appears to be normal in *nsfa*^*I209N*/*st53*^ mutants compared with the null mutants.

To further examine the integrity of the synaptic contacts, we used anti-Ribeye b and anti-MAGUK (membrane-associated guanylate kinase) antibodies to label pre- and postsynaptic structures, respectively (Figures 3A and 3C). We counted the number of ribbons per neuromast (restricted to trunk neuromasts “LL1–2”) in confocal z stacks and did not find any significant difference between WT and *nsfa*^*I209N*/*st53*^ mutants (Figure 3D), nor did we observe a difference in the size of the ribbons (Figure 3E). Colocalization of MAGUK and Ribeye b was also comparable between WT and *nsfa*^*I209N*/*st53*^ larvae (Figure 3F). In contrast, all three features were significantly decreased in the *nsfa* null mutant (Figures 3D–3F). Lastly, we quantified the intensity of the MAGUK immunolabel and did not detect any significant changes in the *nsfa*^*I209N*/*st53*^ mutants (Figure 3G).

Fusion of membrane vesicles is essential to the trafficking of membrane proteins to various subcellular compartments.^30^ Therefore, we investigated whether the I209N mutation has an impact on a key synaptic vesicle protein that is essential for synaptic transmission in zebrafish hair cells, vesicular glutamate transporter 3 (VGlut3).^31^ In null mutants, VGlut3 labeling was dramatically reduced; however, in *nsfa*^*I209N*/*st53*^ mutants the intensity of immunolabel was comparable with that in WT hair cells (Figures 3H and 3I). Collectively, these results indicate that the gross morphology of hair-cell synapses is intact in I209N mutants, in contrast to the deinnervation and reduced levels of crit ical synaptic components in the *nsfa* null mutant.

### Synaptic transmission is impaired in *nsfa*^*I209N*/*st53*^ mutants

Previous studies have shown that the N-D1 linker of the Nsfa protein (Figures 6A and 6B) is critical for SNARE disassembly,^5,32^ yet we did not detect morphological changes in the ribbon synapses of the compound I209N mutant. As opposed to the degeneration seen in the *nsfa* null mutant, we hypothesized that functional deficits in synaptic transmission are causal to the hearing and balance defects in I209N mutants.

The circuitry mediating larval auditory/vestibular reflexes involves several orders of synapses, with the first-order synapse being between hair cells and afferent neurons. As the *nsfa*^*I209N*/*st53*^ mutant has starkly reduced acoustic startle responses and more pronounced vestibular dysfunction than the *nsfa*^*I209N*^ homozygous mutants, we surmised that a defect in synaptic transmission may be present at the first-order synapse of both acoustic and vestibular circuits and, by extension, at the hair-cell synapses in the lateral-line system. Therefore, we utilized an established method to examine action potential spiking in lateral-line afferent neurons in response to mechanical stimulation of lateral-line hair cells.

**Figure 4.**
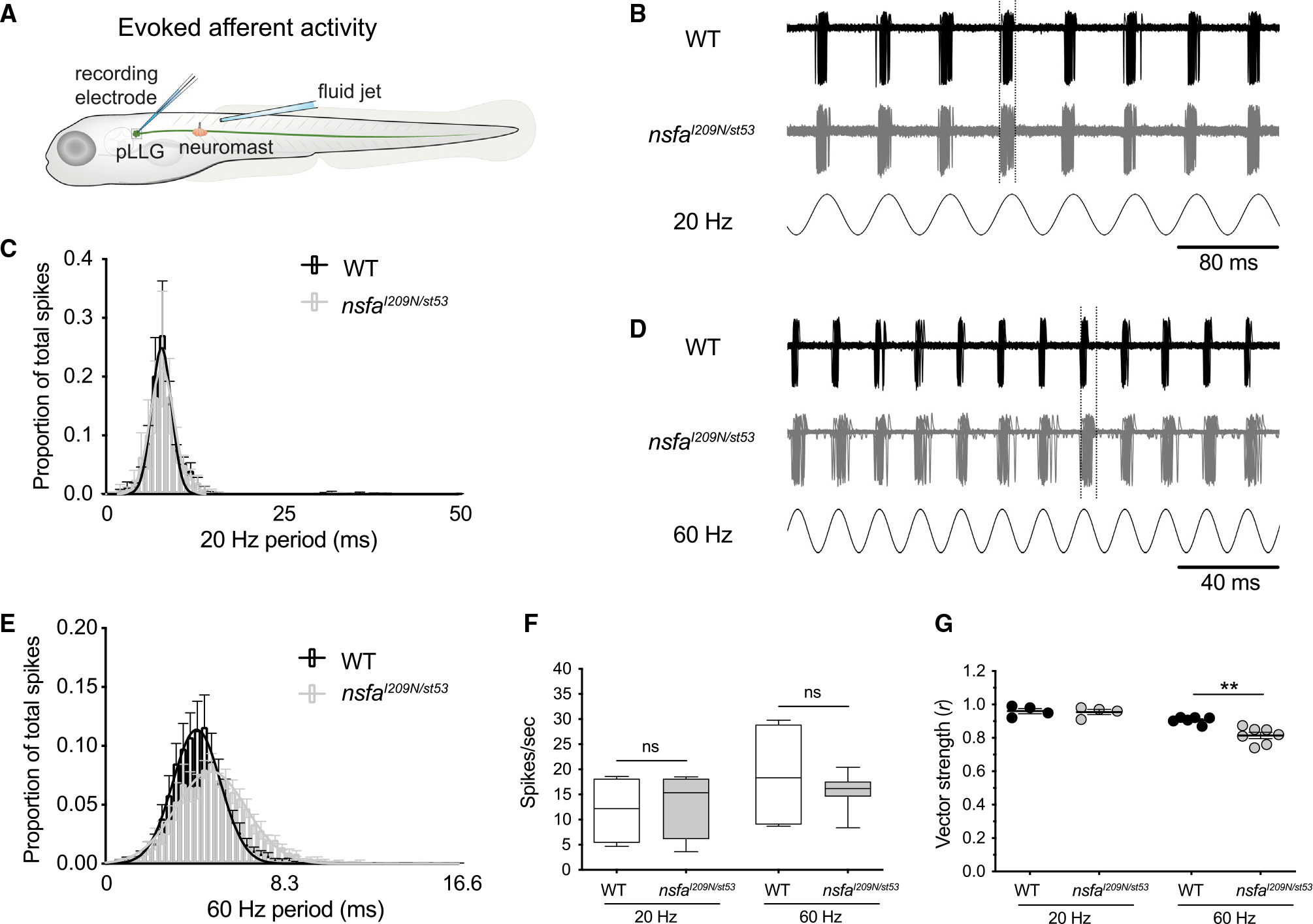
Decreased phase locking to mechanical stimuli at hair-cell ribbon synapses in *nsfa*^*I209N/st53*^ mutants at 60 Hz (A) Schematic of the recording paradigm. Hair cells were mechanically stimulated after establishing a loose-patch recording from the innervating afferent neuron (5 dpf). (B and C) Evoked spike rates and phase locking during a lower-frequency stimulus. (B) Representative traces from WT (upper) and *nsfa*^*I209N/st53*^ mutants (middle) during 20-Hz stimulation (bottom). Shown are 60 overlaid sweeps of spiking resulting in 546 spikes in WT and 559 spikes in *nsfa*^*I209N/st53*^ mutants. (C) Average spike latency histograms from all spikes during 60 continuous seconds of 20-Hz stimulation. WT latency values (black bars) and *nsfa*^*I209N/st53*^ mutant values (gray bars) were fit by a Gaussian distribution (black and gray line, mean fraction of 20-Hz period 0.158 ± 0.002 and 0.159 ± 0.003 s, respectively). (D and E) The timing of evoked spikes is less tightly coupled to a higher-frequency stimulus in the *nsfa*^*I209N/st53*^ mutant. (D) Representative traces from WT (upper) and *nsfa*^*I209N/st53*^ mutants (middle) during 60-Hz stimulation (bottom). Shown are 60 overlaid sweeps of spiking resulting in 219 spikes in WT and 217 spikes in *nsfa*^*I209N/st53*^ mutants. (E) Average latency histograms from all spikes during 60 continuous seconds of 60-Hz stimulation. WT latency values (black bars) and *nsfa*^*I209N/st53*^ values (gray bars) were fit by a Gaussian distribution (black and gray line, mean fraction of 60-Hz period 0.258 ± 0.003 and 0.298 ± 0.003 s, respectively). The peak of activity in mutant neurons is shifted to a later time point of the 60-Hz cycle (4.28 versus 4.95 ms) in comparison. (F) Spike rate comparison between WT and *nsfa*^*I209N/st53*^ mutants at both 20 Hz (WT, 12 ± 3 spikes/s, n = 4; *nsfa*^*I209N/st53*^, 13 ± 3 spikes/s, n = 4, p = 0.80) and 60 Hz (WT, 19 ± 3 spikes/s, n = 7; *nsfa*^*I209N/st53*^, 16 ± 1 spikes/s, n = 7, p = 0.32). Center lines represent the mean. (G) Vector strength (r) of the coupling between stimulus and response (phase locking) between WT and *nsfa*^*I209N/st53*^ mutants at 20 Hz (WT, r = 0.96 ± 0.02, n = 4; *nsfa*^*I209N/st53*^ mutants, r = 0.96 ± 0.02, n = 4) and 60 Hz (from C: WT, r = 0.91 ± 0.01, n = 6; *nsfa*^*I209N/st53*^ mutants, r = 0.81 ± 0.02, n = 7, p = 0.001). p values are determined by unpaired Student’s two-tailed t test. ns, not significant; *p < 0.05, **p < 0.01, ***p < 0.001.

Through loose-patch recordings from the somas of afferent neurons in *nsfa*^*I209N*/*st53*^ mutants (Figure 4A), we examined action currents evoked by a sinusoidal fluid jet stimulation of the hair cells at 20 and 60 Hz (Figures 4B and 4D). We plotted each spike relative to the start of the cycle of the sine wave that evoked the response (Figures 4C and 4E). The distribution of the timing of evoked spikes could be fitted with a Gaussian curve. At both frequencies tested, no significant difference in the number of spikes was observed (Figure 4F), and at 20 Hz the phase locking of afferent spiking to the mechanical stimulus was comparable with WT phase locking (Figures 4B and 4C). In contrast, a slightly delayed and broadened distribution of spiking activity was found in *nsfa*^*I209N*/*st53*^ mutants when the stimulation frequency was increased 3-fold to 60-Hz stimuli (Figures 4D and 4E). Again, the number of spikes evoked by 60-Hz stimuli did not significantly differ between WT and mutant afferent neurons (Figure 4F). The precision of phase locking can also be quantitatively determined by calculating vector strength,^33^ which quantifies the timing synchronization of a response to the phase of the stimulus cycle (see STAR Methods). This analysis revealed that vector strength was significantly reduced for *nsfa*^*I209N/st53*^ larvae at the higher 60-Hz stimulus rate (Figure 4G). In zebrafish, mutation of a different synaptic protein involved in vesicle recycling, Synj1, also affects temporal precision, but additionally results in a reduction in the number of evoked action potentials observed.^25^ However, in contrast to *synj1*^*Q296X*^ mutants, and as mentioned above, we did not see a decrease in spike rate in *nsfa*^*I209N/st53*^ mutants (Figure 4F). These results suggest that the effect of the I209N mutation is on temporal precision of release rather than quantity of release of neurotransmitter.

**Figure 5.**
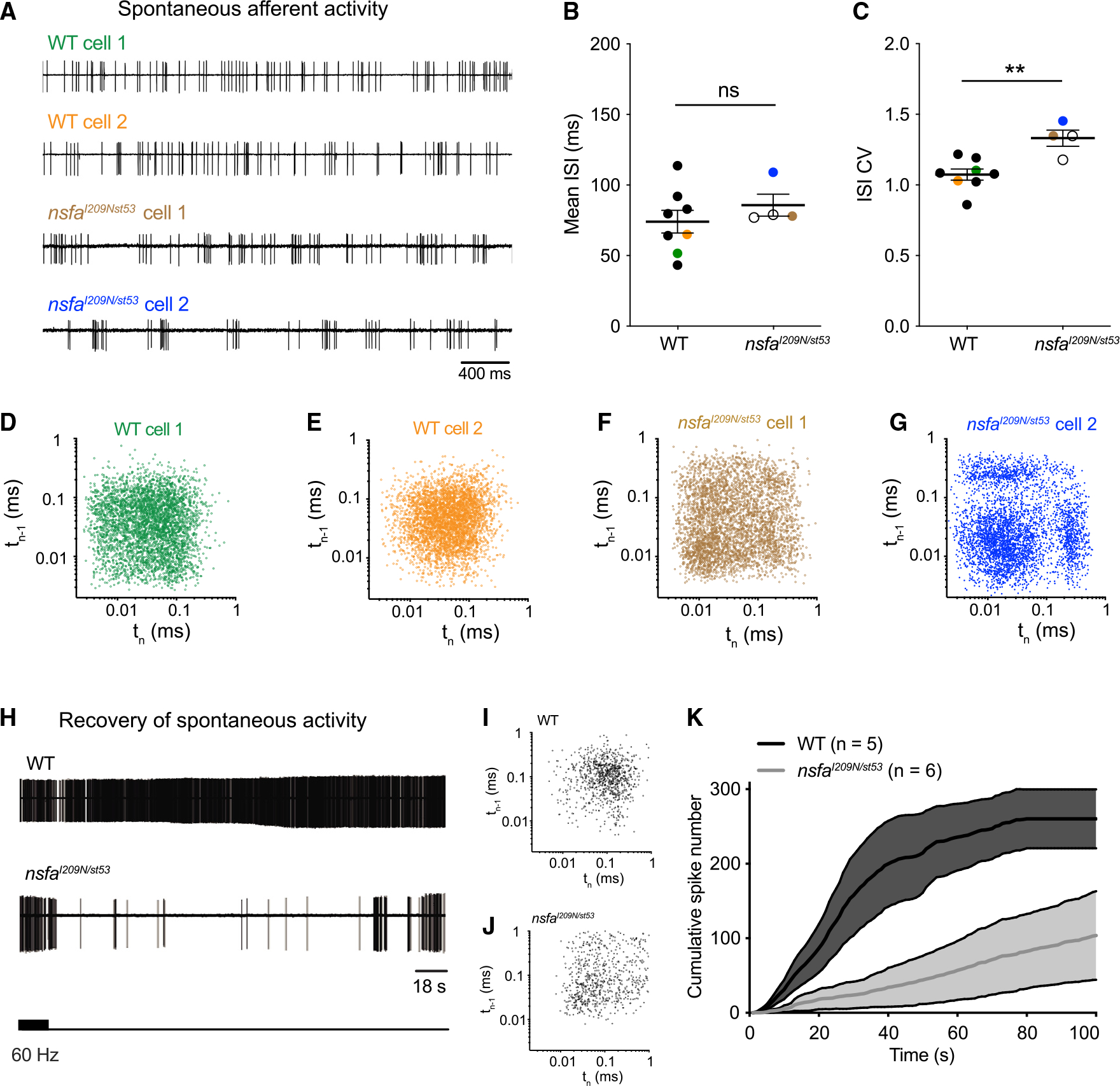
Aberrant timing and decreased recovery of spontaneous activity at hair-cell ribbon synapses in *nsfa*^*I209N/st53*^ mutants (A) Representative traces of spontaneous afferent activity in WT (green and orange traces) and *nsfa*^*I209N/st53*^ mutants (brown and blue traces). (B and C) Mean ISI (B) and ISI CV (C) for spontaneous activity for WT (n = 8 cells) and *nsfa*^*I209N/st53*^ mutants (n = 4 cells). Mean ± SEM are shown; p values were determined by unpaired Student’s two-tailed t tests; *p < 0.05, **p < 0.01, ***p < 0.001. (D–G) Recurrence plots reveal regularity of the timing of consecutive spikes (spike time n plotted versus spike time n – 1) for the four cells shown in (A). Note the greater spread and clustering in three quadrants for the mutant panels, indicating a more irregular, bursting pattern in mutant neurons. (H–K) Recovery of spontaneous activity is delayed in *nsfa*^*I209N/st53*^ mutants. (H) Representative traces show the return of spontaneous activity after a prolonged stimulation of hair cells at 60 Hz. (I and J) Corresponding recurrence plots for the first 900 recovered spontaneous spikes for the recordings shown in (H). (K) Recovery of spontaneous spike rate as shown by cumulative spike number over time following cessation of 90 s of 60-Hz stimulation. Shading represents error. n indicates cell number.

Spontaneous activity of lateral-line afferent neurons is generated by the release of glutamate from the ribbon synapse in the absence of mechanical stimuli.^34^ Given the role of synaptic machinery in triggering hair-cell-evoked spontaneous activity in afferent neurons, we hypothesized that disruption of Nsfa activity would also impact spontaneous action potentials. Two examples of spontaneous spiking are shown for each genotype in Figure 5A. We determined that the mean interspike interval (ISI) between WT and *nsfa*^*I209N/st53*^ neurons was comparable (Figure 5B), indicating that the number of spontaneous spikes was unchanged in the mutants. However, the covariance of the ISI was significantly increased in mutant afferent neurons (Figure 5C), predicting a change in synaptic release of neurotransmitter that impacts the timing of spontaneous spikes.

In addition to the covariance, we noted periods of burst-like patterns in the spike trains of mutants. Therefore, we generated recurrence plots of the timing of consecutive spikes recorded from the cells in Figures 5A, 5D–5G. These plots showed a spread of events with clusters emerging in different quadrants, indicative of a burst-like pattern of spiking on the mutants where (1) short ISI times follow short ISI times within the bursts, (2) short ISI times follow long ISI times, and (3) vice versa for the transitions into and out of burst periods.

Our results examining evoked activity at 60 Hz indicated that temporal defects became apparent with higher-frequency stimulation. Furthermore, our spontaneous data suggested changes in the timing of synaptic release in the absence of hair-cell stimulation. Therefore, we hypothesized that transitioning between prolonged stimulation to spontaneous activity would reveal a further physiological defect. We tested the recovery of spontaneous firing following exposure to a prolonged stimulus. For these experiments, we mechanically stimulated hair cells at 60 Hz for 900 s and recorded the return of spontaneous activity (Figure 5H). For the two examples shown, we saw a similar spread in the recurrence plots for the mutant neuron (Figures 5I and 5J). The cumulative spike number during the course of recovery shows a striking difference in the rate of recovery in the mutants (Figure 5K). Together, these results suggest that the disassembly activity of Nsfa is rate limiting with respect to the basal release of neurotransmitter in hair cells.

### Biochemical characterization of I209N NSF protein

**Figure 6.**
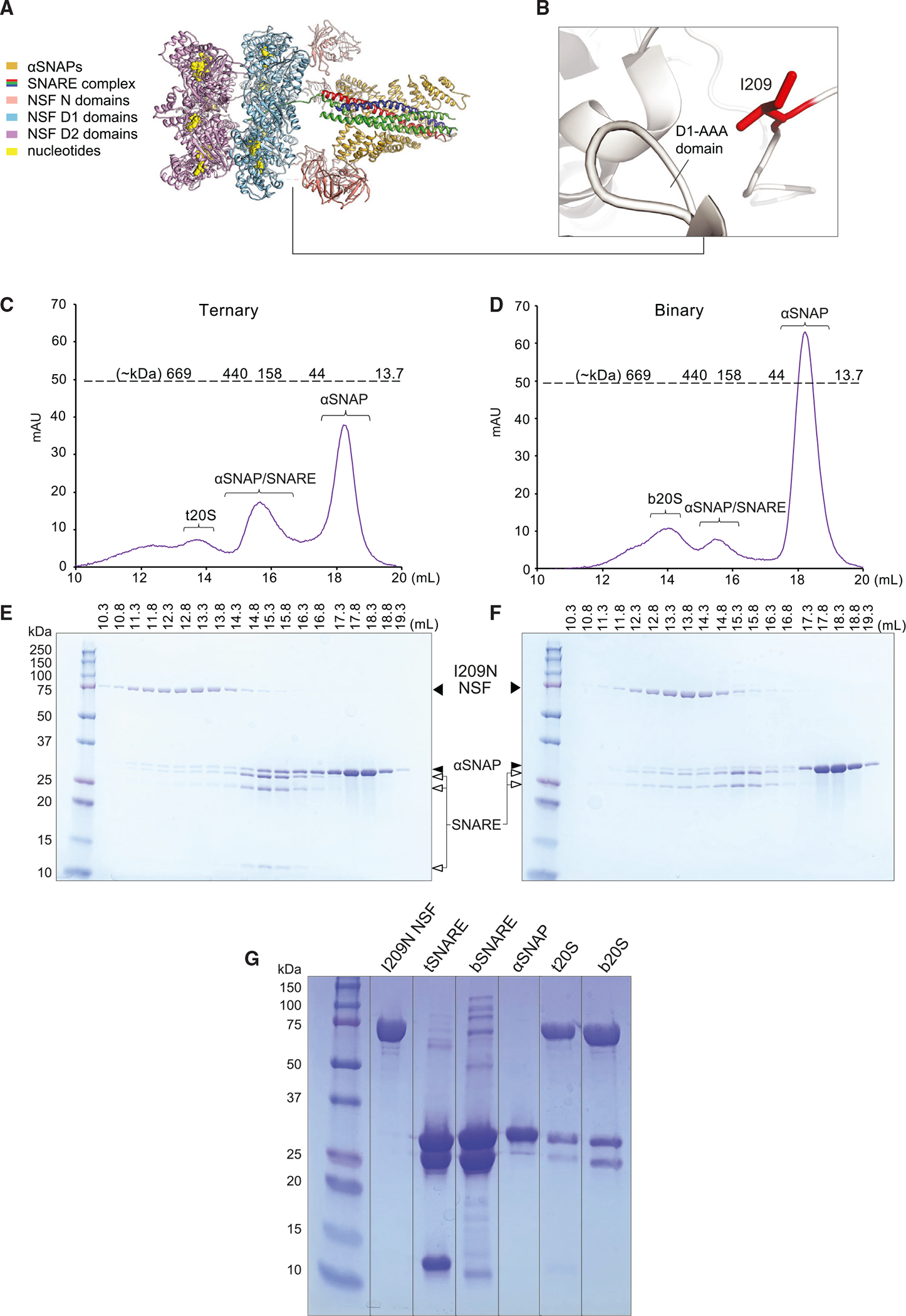
I209N NSF-αSNAP form complexes with either ternary or binary neuronal SNARE complexes (A) Cryo-EM structure of NSF 20S complex (PDB: 6MDM). (B) N-D1 linker region. I209 is shown as a red stick model. (C) Size-exclusion chromatography (SEC) profile of t20S consisting of I209N NSF, ternary (syntaxin-1A-SNAP-25-synaptobrevin-2) SNARE complex (tSNARE), and αSNAP mixed in 1:5:25 M ratios. Predicted molecular weights for the SEC are indicated above the dotted line. (D) SEC of b20S consisting of I209N NSF, binary (syntaxin-1A-SNAP-25) SNARE complex (bSNARE), and αSNAP mixed in 1:5:25 M ratios. (E) SDS-PAGE gel of fractions from SEC of t20S. The elution volumes are indicated above the gel. (F) SDS-PAGE of protein ladder and gel fractions from SEC of b20S. (G) SDS-PAGE gel of protein ladder, NSF stock, tSNARE, bSNARE, αSNAP, t20S filtered through a 100k MWCO concentrator, and b20S filtered through a 100k MWCO concentrator. SEC peaks are typical of a 20S complex preparation with WT NSF.^5^ The sizes of the protein species are as follows: I209N NSF, 82.8 kDa monomeric and 496.8 kDa hexameric; αSNAP, 33.3 kDa; ternary SNARE complex, 65.2 kDa; binary SNARE complex, 54.4 kDa.

To gain more insight into the effect of the I209N mutation on the activity of NSF, we utilized a biochemical approach, generating the same mutation in the *C*. *griseus* NSF ortholog for our experiments. Based on high-resolution cryo-EM structures,^6^ isoleucine 209 directly follows the linker region between NSF’s N domain and its D1 AAA^+^ domain (Figures 6A and 6B). Structurally, the side chain is buried between several other hydrophobic residues, where it is predicted to make multiple inter- and intraprotomer stabilizing hydrophobic interactions.^6^ Mutation from isoleucine to asparagine likely precludes these interactions, effectively increasing the N-D1 linker length, thereby increasing N-domain motility.

To determine whether the I209N protein is able to form the active complex, we produced high-purity protein as described previously^5^ and tested NSF I209N’s ability to form complexes consisting of NSF, αSNAP, and SNARE complexes. For these experiments, we examined both the ternary and binary SNARE complex. The so-called ternary 20S complex (t20S) was formed by preparing the αSNAP-SNARE complex and then mixing with hexameric NSF I209N in a final ratio of 1:5:25 (NSF I209N/SNARE/αSNAP). The t20S complex was then separated from the individual components by size-exclusion chromatography (SEC); the first species to elute is consistent with t20S complex based on elution volume and SDS-PAGE (Figures 6C, 6E, and 6G). This is consistent with t20S formation with WT NSF.^5^ The so-called binary 20S complex (b20S) was also formed with components mixed in a 1:5:25 ratio (NSF I209N/SNARE complex/αSNAP) and subjected to SEC and SDS-PAGE (Figures 6D, 6F, and 6G). The b20S complex elutes at a volume similar to that of t20S, demonstrating that the NSF I209N mutant is also able to form a complex with the binary SNARE complex. Together, these results suggest that the I209N mutation of NSF does not disrupt NSF hexamerization or substrate recognition and binding.

### I209N NSF differentially affects binary and ternary SNARE complex disassembly

**Figure 7.**
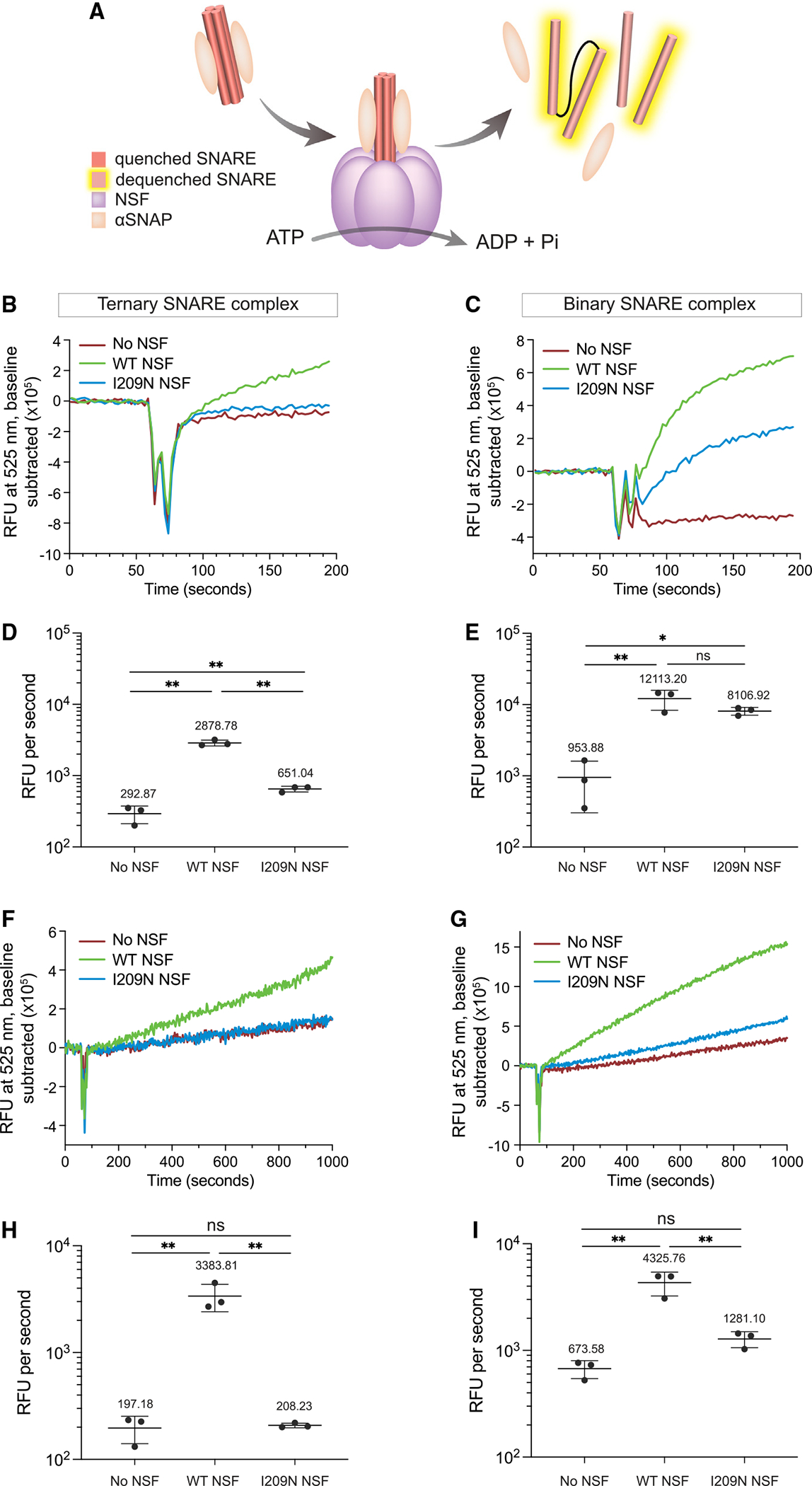
Kinetic traces of I209N NSF show diminished ability to disassemble ternary SNARE complex but retention of binary SNARE complex disassembly activity (A) Schematic of NSF-mediated disassembly. NSF (purple), αSNAP (beige), and SNARE helices are depicted. (B) NSF-mediated disassembly of ternary complex. [NSF] = 42.2 nM. (C) NSF-mediated disassembly of binary complex. [NSF] = 42.2 nM. (D) Initial rate of NSF-mediated disassembly of ternary complex with NSF. [NSF] = 42.2 nM. (E) Initial rate of NSF-mediated disassembly of binary complex with NSF. [NSF] = 42.2 nM. (F) NSF-mediated disassembly of ternary complex. [NSF] = 8.3 nM. (G) NSF-mediated disassembly of binary complex. [NSF] = 8.3 nM. (H) Initial rate of NSF-mediated disassembly of ternary complex with NSF. [NSF] = 8.3 nM. (I) Initial rate of NSF-mediated disassembly of binary complex with NSF. [NSF] = 8.3 nM. All experiments are n = 3. Error bars in (B), (C), (F), and (G) are SEM of the three independent experiments. Error bars in (D), (E), (H), and (I) are SE from Python package SciPy linear regression. p values in (D), (E), (H), and (I) are derived from one-way ANOVA. ns, not significant; *p < 0.05, **p < 0.01.

We next assessed the competence of I209N NSF in ternary SNARE complex disassembly as described previously.^5,35^ In brief, multiple cysteine residues on the different SNARE proteins are labeled with Oregon green 488 maleimide, and dye fluorescence is quenched upon SNARE complex formation due to dye proximity. The fluorophores are dequenched upon disassembly by NSF, leading to an increase in detected fluorescence (Figure 7A). Kinetic traces of the disassembly activity without NSF, with NSF, and with I209N NSF (Figure 7B) show that the disassembly activity of ternary complexes by I209N NSF is substantially slower (Figure 7D).

We then developed an assay to monitor binary (SNAP-25-syntaxin-1A) SNARE complex disassembly; in short, we assembled SNARE complex with only SNAP-25 and syntaxin-1A with the same cysteine residues introduced and labeled as in the ternary complex disassembly assay. Previous single-molecule fluorescence resonance energy transfer studies showed disassembly activity of the binary SNARE complex to be substantially faster than that of the ternary complex.^36^ Our disassembly assay confirms this result; the binary complex is disassembled substantially faster than the ternary complex by WT NSF (Figures 7B–7E). Strikingly, I209N NSF disassembles binary SNARE complex more efficiently than the ternary SNARE complex at the concentration used in Figures 7B–7E. Initial rates of disassembly were not significantly different (Figure 7E). Nevertheless, the binary complex disassembly activity of I209N NSF is slightly lower than that of WT NSF.

To further compare the rate of binary complex disassembly by WT and I209N NSF and assess the dependence on NSF concentration, the above experiments were repeated with ~5-fold less NSF. Under these conditions, I209N NSF produces no measurable ternary SNARE complex disassembly (Figure 7F) and disassembles the binary SNARE complex substantially slower than WT NSF (Figure 7G). At lower WT NSF concentration, the rate of binary complex disassembly is statistically significantly higher than with I209N NSF (Figure 7I). Taken together, I209N NSF is able to disassemble binary SNARE complexes at the NSF concentration used in Figure 7F–7I,^3,4^ but its ternary complex disassembly activity is greatly impaired.

## DISCUSSION

NSF and SNAP are required for quality control and recycling of SNARE complexes that mediate membrane fusion.^3,4,37^ Here, we elucidate the effects of a hypomorphic allele of NSF on SNARE disassembly and sensory hair-cell function in zebrafish larvae. In contrast to previously identified truncating mutations of *nsfa*, this hypomorphic allele results in the substitution of a polar amino acid (asparagine) for a hydrophobic isoleucine residue at position 209. In protein structures of NSF, the I209 side chain is buried within a hydrophobic pocket at the end of the linker region, directly adjacent to the catalytic ATPase domain.^6^ Our *in vivo* and *in vitro* experiments reveal that the I209N substitution causes selective effects on NSF function and disassembly activity. We found that the I209N mutation results in temporal deficits in synaptic transmission at hair-cell synapses without an accompanying loss of myelination of the afferent neurons or deinnervation of hair cells that is prominent in *nsfa* null mutants. Consistent with the hypomorphic phenotype, *in vitro* analysis of I209N NSF function revealed a differential effect on SNARE complex disassembly. Although hexamer formation and substrate recognition are comparable with those of WT NSF, the I209N mutation shows differences in binary (syntaxin-1-SNAP-25) versus ternary (syntaxin-1-SNAP-25-synaptobrevin-2) SNARE complex disassembly activity in a concentration-dependent manner. Disassembly assays with concentrations of NSF used in previous studies^35^(Figures 7B–7E) demonstrate that the I209N protein substantially slows ternary SNARE complex disassembly but only modestly reduces binary SNARE complex disassembly. At approximately 5-fold lower concentrations, binary complex disassembly is strongly reduced, whereas ternary complex disassembly is almost absent (Figures 7F–7I). The concentration-dependent effect on *in vitro* SNARE disassembly is consistent with the gene-dosage effect on auditory/vestibular behavioral reflexes observed with the I209N allele. Sensitivity to rotary stimuli and high-intensity tones are more compromised in larvae carrying one copy of the *I209N* allele in a null background compared with mutants expressing two copies of *I209N* allele. Combined, our analyses of the selective effects of the I209N mutation on NSF activity reveal concentration-dependent effects that can impact sensory behavior.

The selective *in vivo* deficits caused by loss of *nsfa* function are due in part to the duplication of the *nsf* gene in zebrafish and the developmental delay in the onset of the defects after maternal transcripts and proteins decline, similar to a conditional knockout. Due to normal early development during the embryo-to-larva transition, the I209N hypomorph provided an opportunity to dissect the role of Nsfa in membrane fusion in a particular cell type, the hair cell. Both the normal innervation of I209N mutant hair cells, which relies on the secretion of neurotrophic factors such as Bdnf from hair cells,^13,38^ and the abundance of the integral membrane protein VGlut3 in synaptic vesicles in I209N mutant hair cells at later larval stages, suggest that intracellular membrane trafficking is unaffected in the I209N mutant. In contrast, the *nsfa* null mutant shows obvious hallmarks of defective membrane trafficking and degeneration in hair cells, including deinnervation, fewer and smaller ribbons, and lower levels of VGlut3. These data highlight the hypomorphic nature of the I209N mutation with respect to developmental processes that require membrane trafficking and vesicle secretion. Our study also suggests that quality control by Nsfa, that is, the disassembly of off-target binary SNARE complexes, is key to a variety of neurodevelopmental processes and maintenance of synaptic contacts.

The normal morphology of the synapses in lateral-line hair cells combined with the auditory and vestibular behavioral deficits in the hypomorphic mutant suggests that the I209N mutation specifically affects synaptic transmission. Consistent with this idea, our recordings of afferent nerve activity in the lateral-line system demonstrate that temporal changes in spiking activity occur when mutant hair cells are stimulated by mechanical stimuli. Impairment of the timing of evoked afferent nerve activity manifests as a delay in the timing of spiking and a less tuned or broadened period during which spiking occurs in response to a mechanical stimulus. These temporal impairments in neural activity, however, were only detectable in the I209N mutant when using a higher-frequency stimulus. We surmise that the partial function of the I209N protein in binary SNARE complex disassembly can provide sufficient priming of the exocytic machinery during lower-frequency stimulation of hair cells. Nevertheless, irrespective of stimulus frequency, the evoked number of afferent spikes was not reduced in the I209N mutant. Unlike central synapses, hair-cell synapses contain large pools of synaptic vesicles.^27,39,40^ Disassembly and recycling of ternary SNARE complexes may not be the rate-limiting step in hair-cell exocytosis unless the large pool of releasable synaptic vesicles is depleted. Indeed, the most striking deficit at hair-cell synapses in the I209N mutant is seen in the recovery of spontaneous release of neurotransmitter after exposure to prolonged, high-frequency mechanical stimulation. The return of spontaneous spiking of afferent neurons is greatly delayed and resumes at a much slower rate in the I209N mutant. In the absence of SNARE ternary disassembly, the rundown of hair-cell synaptic vesicles in the I209N mutant may shift the dependence of the system to recycling of the SNARE exocytic machinery as the rate-limiting step. That spontaneous release does eventually resume at a reduced rate in the I209N mutant suggests that disassembly of ternary SNARE complexes is still possible, albeit at very low levels given the pronounced reduction of ternary SNARE disassembly *in vitro*. Alternatively, renewal of the fusogenic pools of SNARE complexes via binary SNARE disassembly^3,4^ and/or regeneration of the synaptic vesicle pool may occur within minutes of rundown, slowly leading back to the normal rates of spontaneous release seen at rest in the I209N mutant. Although the rate of spontaneous release by mutant hair cells is comparable with that of the WT at rest, we also noted a temporal difference in spontaneous activity. Unlike WT afferent neurons, I209N mutant afferents exhibit a burst-like pattern of spontaneous activity. Why the ISIs vary in the I209N mutant is not clear and requires further investigation.

What is the biochemical origin of the selective effects on binary versus ternary SNARE disassembly and the resulting consequences *in vivo*? While the ternary SNARE complex is exceptionally stable, binary SNARE complexes are much less so.^41^ The differential effect of the I209N mutation on disassembly of ternary and binary SNARE complexes is thus consistent with an effect on the application of force by NSF and SNAP to substrate. More specifically, such an effect could be the result of a reduction in peak force, a reduction in force integrated over time, a reduction in intersubunit coordination, or some combination of all three. While the specific molecular mechanism by which NSF disassembles the SNARE complex remains to be uncovered, one could envision that the I209N mutation affects N-domain motility.^5^

In sum, our study offers insight into the selective effects of the I209N mutation in *nsfa* on neurodevelopment and function in zebrafish. The unique effect of the mutation allowed us to compare and contrast the *in vivo* consequences of differential effects on preand postfusion disassembly of SNARE complexes. The ability of I209N NSF to disassemble the binary SNARE complex, albeit modestly reduced at the higher concentration that we tested, paired with a severe reduction in its ability to disassembly ternary SNARE complexes *in vitro*, provides clues about the nature of the relatively mild phenotype of I209N mutants in contrast to the null condition. Our results suggest that disassembly of the binary SNARE complex is more critical than previously appreciated, placing quality control of nonfusogenic binary SNARE complexes on par with ternary SNARE complex disassembly in terms of importance to normal development and maintenance of the nervous system.

### Limitations of the study

Our study included analysis of synaptic transmission at the hair-cell synapses of the lateral-line system; however, synaptic transmission deficits may also exist at other types of synapses of the acoustic startle reflex or VSR circuits as *nsfa* is ubiquitously expressed in the nervous system. In addition, we did not analyze the ultrastructure of hair-cell synapses or higher-order synapses of the acousticolateralis system in this study. It is a possibility that subtle fine structural changes resulting from membrane-trafficking defects could also partly account for the phenotype of the I209N mutant.

The gene-dosage effect that we observed in *I209N* mutants depended on the sensory circuit and conditions tested, that is, the copy number of the *I209N* mutation resulted in a more profound difference in the VSR, whereas the acoustic startle reflex was strongly reduced irrespective of copy number. A statistically significant difference between mutant genotypes only emerged at the highest intensity of sound stimulation. These findings suggest that certain synapses and/or circuits are more sensitive to the *I209N* mutation regardless of mutant Nsfa protein levels. Sensitivity may in part be determined by a heavier reliance on SNARE complex recycling for continual exocytosis of synaptic vesicles. In contrast to the sensory deficits in the *I209N* mutant, the function of the motor system is grossly unaffected. Nevertheless, future in-depth analyses of swimming behaviors may also reveal more subtle changes in motor activity, especially under conditions where synaptic vesicle turnover is increased.

## STAR★METHODS

### RESOURCE AVAILABILITY

#### Lead contact

Further information and requests for resources and reagents should be directed to and will be fulfilled by the lead contact, Teresa Nicolson (tnicolso@stanford.edu).

#### Materials availability

The study did not generate new unique reagents. Fish lines used in this paper will be shared freely upon request to the lead contact.

#### Data and code availability

- Original data are available from the lead contact upon request.
- This paper does not report original code.
- Any additional information in this work is available from the lead contact upon request.

### EXPERIMENTAL MODEL AND SUBJECT DETAILS

#### Zebrafish husbandry

WT, mutant, and transgenic strains were maintained in a Tübingen or Top Long Fin background as described.^42^ Larvae were raised at 28.5 °C in the dark in E3 buffer. The *nsfa*^*st53*^ allele was obtained from the Zebrafish International Resource Center; the *nsfa*^*I209N*^ allele was identified in a large-scale genetic screen (Tübingen 2000) at the Max Planck Institute for Developmental Biology, Tübingen, Germany. Experiments were performed with the approval of the Stanford University, Amherst College, and the Oregon Health and Science University Institutional Animal Care and Use Committee and in accordance with NIH guidelines.

### METHOD DETAILS

#### Positional cloning of*nsfa*^*I209N*^

Themutation was genetically mapped using segregation analysis with PCR-based simple sequence length polymorphisms (SSLPs; z9964, z25578, BX890590, BX890565, and CR387987).^43^ To determine the mutation in *nsfa* gene, exons from the *nsf*a region of mutant and WT sibling embryos were amplified and sequenced. Primers to amply exon 8 (F: GGT CGG CCT GTT GGT TGG AAA CAG TCA AGT; R: CAT CTG CTC CAC AAT GTC TGG AGGAAA) were used for genotyping.

#### Quantitative PCR

mRNA was extracted from 5 dpf zebrafish larvae using TRIzol (Thermo Fisher) and RNeasy kit (Qiagen). mRNA samples from different resources were then adjusted to equal concentration before reverse transcription. SuperScript III Platinum Two-Step qRT-PCT kit was used to reverse transcribe 1 μg RNA to cDNA (Invitrogen). 1 μl of 1:20 dilution of cDNA and 10μl SYBR green mixtures were used for each reaction in a 96-well plate on an Applied Biosystems 7900 HT real-time PCR machine. Expression levels of genes were calculated from a cDNA standard curve and then normalized to the reference gene *gapdh* RNA level. Sequences used for qPCR are: *nsfa* (F: CGT GGT TGA TGA CAT TGA GC; R: CGA CCA TGA GGT GGA GTC TT), *nsfb* (F: CAT GTC CGA CTC TTT CAG CA; R: CCT TTC ACC TGT TTG CCA AT), *gapdh* (F: GTG GAG TCT ACT GGT GTC TTC; R: GTG CAG GAG GCA TTG CTT ACA).

#### Behavioral responses

Measurement of the auditory evoked behavioral response (AEBR) and recording of the behavior was adopted from previous publication with minor changes.^26^ Briefly, 5 dpf zebrafish larvae in a 96-well microplate were stimulated by a mini-shaker (type 4810, Bruel & Kjaer) in the dark and recorded using a Zebrabox monitoring system (ViewPoint Life Sciences). The sound pressure level (SPL) was pre-determined using a hydrophone (WP-23502-P16, Knowles Electronics) and an oscilloscope (TDS 1002B, Tektronix) before testing. Trials of 100 ms stimuli were performed at the chosen intensities at 600 Hz; each trial was separated by 5 minutes resting periods. For each larva, three trials were performed at each frequency and two trials with the best response were selected to calculate the percentage of responses.

Details of VSR tests were described previously.^29^ Briefly, the 5 dpf zebrafish larval head was immobilized in 2% agarose, and the trunk and pectoral fins were free to move in the E3 buffer. The device provided a platform rotation of ±75° and a sinusoidal stimulation at 0.53 Hz. The VSR videos were analyzed by ZebraZoom program.^44^ Output data were processed using the GraphPad Prism 9. Mean ± SEM and two-way ANOVA with Benjamini-Hochberg correction for each dataset were performed.

#### Immunofluorescence labeling and imaging

Antibodies and their staining protocols used in this research were described previously.^13^ Briefly, after 5 dpf zebrafish larvae were fixed in PBS buffer supplied by 4% PFA/4% Sucrose/0.01% Tween-20 overnight at 4° C, they were then permeabilized with acetone for 7 minutes at −20°C and blocked in PBS containing 2% fish gelatin/1% BSA/1% goat serum/1% DMSO (FGBS). After blocking, specimens were incubated with primary antibodies in FGBS overnight at 4° C (1:100 anti-NSF, Cell Signaling Technology; 1: 4000 anti-Ribeye b, Openbiosystems; 1:500 anti-zn12/HNK-1, Zebrafish International Resource Center; 1:500 anti-pan MAGUK, UC Davis/NIH NeuroMab Facility; 1: 1000 Vglut3, Proteintech; 1:50 anti-Mbp, gift from W. Talbot; 1:1500 anti-Acetylated tubulin, Sigma; 1:1000 anti-FIGQY, gift from M. Rasband), followed by incubation with appropriate secondary antibodies (1:1500 conjugated to Alexa 488 or Alexa 568, Invitrogen) in FGBS overnight at 4° C. A Zeiss Axiovert ImagerM.1 microscope with an LSM700 confocal scanhead, Axiocam MrM camera, and water-immersion lens Zeiss Plan Apochromat 63X/1.4NA objective (Zeiss) were used to take Z-stack images of the lateral line nerve and neuromasts.

#### Electrophysiology

The recording setup for spontaneous and evoked action currents was described previously.^45,46^ Briefly, larvae were paralyzed by first immersing in the anesthetic (MS-222; Sigma) and then with microinjection into the heart with 125 μM α-bungarotoxin. Larvae were then rinsed and submerged in extracellular solution (in mM: 130 NaCl, 2 KCl, 2 CaCl_2_, 1 MgCl_2_ and 10 HEPES; 310 mOsm; pH 7.8). For extracellular action current recordings, borosilicate glass pipettes were pulled to a long taper with resistances between 5 and 15 MΩ in extracellular solution. Action currents were acquired from a loose-patch of the cell body of an individual lateral-line afferent neuron (seal resistances ranged from 20 to 80 MΩ). Recordings were collected using an EPC 10 amplifier and Patchmaster software (Heka Electronic, NY) in voltage-clamp mode (sampled at 50 μs/pt; filtered at 1 kHz). Each recording is obtained from one cell from a single larva.

#### Mechanical stimulation

Stimulation of hair cells was performed as described previously.^45^ In short, a glass micropipette of ~30 μm tip diameter filled with normal extracellular solution was attached to a HSPC-1 pressure clamp (ALA Scientific, NY). The HSPC-1 was driven by analog output from the data acquisition board. The fluidjet micropipette was positioned via a MP-265 micromanipulator (Sutter Instruments, CA) approximately 100 μm from a chosen neuromast and displacement of the cupula and kinocilia was verified by observation. Fluid-jet pressure was monitored from the analog output of a feedback sensor located on the headstage of the HSPC-1 and data were collected alongside the action current recording for each experiment.

#### Signal analysis

Data were analyzed using custom routines^45^ in Igor Pro (Wavemetrics, Lake Oswego, OR) and were plotted with Adobe Illustrator and Prism 9. Histograms were constructed from individual spike times that were normalized to t = 0 for each sine-wave period (20 Hz = 50 ms and 60 Hz ≈ 16.6 ms). The degree of synchrony across the latency values for each stimulus frequency was then quantified using circular statistics. Briefly, for the 20 Hz and 60 Hz stimulus, each latency value was converted to a unit vector (length = 1) of specific phase angle. Then trigonometry was then used to determine the x and y lengths of each unit vector, which were used to calculate the length of the mean vector^33^ that was reported in the text as vector strength (r). For the 20 Hz and 60 Hz stimulus, we converted each latency value to a unit vector of specific phase angle. Then the synchronization index was determined by calculating the length of the mean vector, which is reported as vector strength.^33^ Spontaneous data represent spikes collected from 400 consecutive 1-second sweeps without stimulation. Interspike interval histograms were best-fit (Extra sum-of-squares F test) using either a one-phase or two-phase exponential equation.^34^ Values in the text are expressed as mean ± SEM. Statistical significance of differences between means were determined by either paired or unpaired, two-tailed Student’s t-tests, as appropriate.

#### Purification and reconstitution of NSF, αSNAP, and SNARE complexes

Mutant (I209N) NSF was generated through Agilent QuikChange II Site-directed mutagenesis on the WT *C*. *griseus* sequence. WT and mutant NSF was expressed in BL21(DE3)-RIL *E*. *coli* and purified with Nickel-NTA chromatography, followed by size exclusion chromatography (SEC) on a Superdex 200 16/60 column (GE Healthcare). Hexameric NSF was monomerized by removing ATP from the buffer and replacing it sodium phosphate overnight. Monomeric NSF was purified with SEC and then reassembled by a final SEC run with buffer containing ATP at 1mM.^5^ Rat αSNAP was purified using Nickel-NTA chromatography followed by SEC.^35^ Soluble ternary neuronal SNARE complex consisting of WT SNAP-25A (residues 1–265), S249C/K253C syntaxin-1A and His-tagged synaptobrevin-2 (residues 1–96) were purified using Nickel-NTA chromatography followed by SEC with a Superdex 200 10/300 column.^35^ Soluble binary neuronal SNARE complex consisting of His-tagged WT SNAP-25A (residues 1–265) and S249C/K253C syntaxin-1A was purified in a manner similar to that of the ternary complex, with the exception that the Ni-NTA wash buffer did not include 7.5M Urea.

#### Size exclusion chromatography and 20S formation

NSF, SNARE complexes (ternary or binary), and αSNAP were combined in 1:5:25 molar ratios to form 20S complex. 0.5 nanomoles of NSF, 2.5 nanomoles of SNARE, and 12.5 nanomoles of αSNAP were mixed in 500 microliters 20S degassed buffer (50 mM Tris pH 8, 150 mM NaCl, 1 mM TCEP, 1 mM ATP, 1 mM EDTA). The mixture was then injected immediately onto a Superdex 200 16/60 column (GE Healthcare) that was already equilibrated in 20S buffer. Concentrate from the center of the 20S peak was filtered through a 0.5 mL 100k MWCO filter.

#### Disassembly assays

Both ternary and binary neuronal SNARE complex was labeled overnight in degassed buffer with Oregon Green 488 Maleimide.^35^ Disassembly assays were performed using a FlexStation II 384-well plate reader (Molecular Devices) with a final reaction volume of 60 microliters^6^ with the notable changes being that the NSF final concentration was either 8.3 nM or 42.2 nM, the final labeled SNARE concentration in the reaction was 1720 nM, and the final αSNAP concentration was 1.96 μM after trituration to the final volume of 60 μL. Initial disassembly rates were calculated using the first 112.5 seconds (Ternary) or 222.5 seconds (Binary) after trituration with initiation solution. Statistical significance was calculated using Student’s two-tailed t test.

### QUANTIFICATION AND STATISTICAL ANALYSIS

#### Image analysis

Raw data were processed using Image J and Adobe Illustrator 2022. Quantification of immunofluorescence intensity, particle analysis, and melanophore expansion was performed using ImageJ and Just Another Colocalization Plugin (JACOP) using maximum projections of the lateral line nerve or neuromasts. Analyses were performed blinded to the scorer. For analysis of afferent nerve labeling, an identical ROI was selected in each Z-stack image containing an entire cross section of the nerve to quantify the level of fluorescence. For analysis of ribbon synapses and hair-cell immunofluorescence, Z stacks containing the entire neuromast were quantified. Ribbon bodies within a neuromast were counted manually. Fluorescent signals containing ≥ 20 pixels with three-fold intensity above background were counted as ribbons.

#### Statistical analysis

GraphPad Prism 9.3.1 was used to analyze all data. All values are presented as means ± standard error. P values were determined by paired or unpaired Student’s two-tailed t-tests, or one-way or two-way ANOVA as appropriate.

## Supplementary Material

1

## Figures and Tables

**KEY RESOURCES TABLE:** 

REAGENT or RESOURCE	SOURCE	IDENTIFIER

**Antibodies**		

NSF (1: 100)	Cell Signaling Technology	Cat# 3924; RRID:AB_2155693
Ribeye b (1: 4000)	Generated by Openbiosystems	N/A
HNK(1: 500)	ZIRC, Zn-12	Antibody ID: ZDB-ATB-081002-12
MAGUK(1: 500)	UC Davis/NIH NeuroMab Facility	Cat# 73-029; RRID:AB_2877192
FIGQY (1:1000)	Gift from M. Rasband	N/A
Vglut3 (1: 1000)	Generated by Proteintech	N/A
MBP (1: 50)	Generated by Talbot Lab	N/A
Acetylated Tubulin (1: 1500)	Sigma-Aldrich	Cat# T6793; RRID:AB_477585

**Chemicals**		

TRIzol	Invitrogen	Cat# 15596026
Chloroform	Sigma-Aldrich	Cat# 319988
Isopropanol	Sigma-Aldrich	Cat# 190764
Paraformaldehyde 32%	EMS	Cat# 15714
Low-melting Point Agarose	Sigma-Aldrich	Cat# A4018
DMSO	Sigma-Aldrich	Cat# D2650
Tricaine (MS-222)	Western Chemical Inc.	Cat# TRIC-M-GR-0010
Sylgard 184 Silicone Elastomer Kit	Dow-Corning	https://www.dow.com/en-us/pdp.sylgard-184-silicone-elastomer-kit.01064291z.html
Alpha-bungarotoxin	Sigma-Aldrich	Cat# 203980
Oregon Green^™^ 488 Maleimide	Invitrogen	Cat# O6034

**Critical commercial assays**		

SuperScript III Platinum Two-Step qRT-PCR Kit	Invitrogen	Cat# 11735-032
RNeasy Mini Kit	QIAGEN	Cat# 74104

**Oligonucleotides**		

*nsfa* Genotyping Primers	This paper	N/A
F: GGT CGG CCT GTT GGT TGG AAA CAG TCA AGT; R: CAT CTG CTC CAC AAT GTC TGG AGGAAA		
*nsfa* QPCR Primers	This paper	N/A
F: CGT GGT TGA TGA CAT TGA GC; R: CGA CCA TGA GGT GGA GTC TT		
*nsfb* QPCR Primers	This paper	N/A
F: CAT GTC CGA CTC TTT CAG CA; R: CCT TTC ACC TGT TTG CCA AT		
*gapdh* QPCR Primers	This paper	N/A
F: GTG GAG TCT ACT GGT GTC TTC; R: GTG CAG GAG GCA TTG CTT ACA		

**Software and algorithms**		

GraphPad Prism	Prism_Version 9.3.1	https://www.graphpad.com/scientific-software/prism/
ImageJ	Fiji	RRID:SCR_002285 https://fiji.sc
Black Zen	Zeiss	RRID:SCR_018163 https://www.zeiss.com/microscopy/en/products/software/zeiss-zen.html
Adobe Illustrator 2022	Adobe	https://www.adobe.com/products/illustrator.html
SutterPatch	Sutter Instruments	https://www.sutter.com/AMPLIFIERS/SutterPatch.html
Python version 3.8	Python Software Foundation	https://www.python.org/downloads/release/python-380/

**Other**		

FLEXstation II 384	Molecular Devices	N/A

## Highlights

- Hypomorphic *nsf* mutation causes selective effects on sensorineural development and function
- I209N mutation causes reduced precision and recovery of activity at hair-cell synapses
- I209N mutation has differential effects on pre- and postfusion SNARE complex disassembly
- Quality control of binary SNARE complexes is critical for neurodevelopment
