# Supplementary material for: Sensory deficit screen identifies nsf mutation that differentially affects SNARE recycling and quality control
Source: Cell Rep. Author manuscript; Available in PMC 2023 Sep 27. (PMC10524599; doi:10.1016/j.celrep.2023.112345)
Supplement: 1 [file NIHMS1895586-supplement-1.pdf]

Cell Reports, Volume *42*

Supplemental information

Sensory deficit screen identifies *nsf* mutation that differentially affects SNARE recycling and quality control

Yan Gao, Yousuf A. Khan, Weike Mo, K. Ian White, Matthew Perkins, Richard A. Pfuetzner, Josef G. Trapani, Axel T. Brunger, and Teresa Nicolson

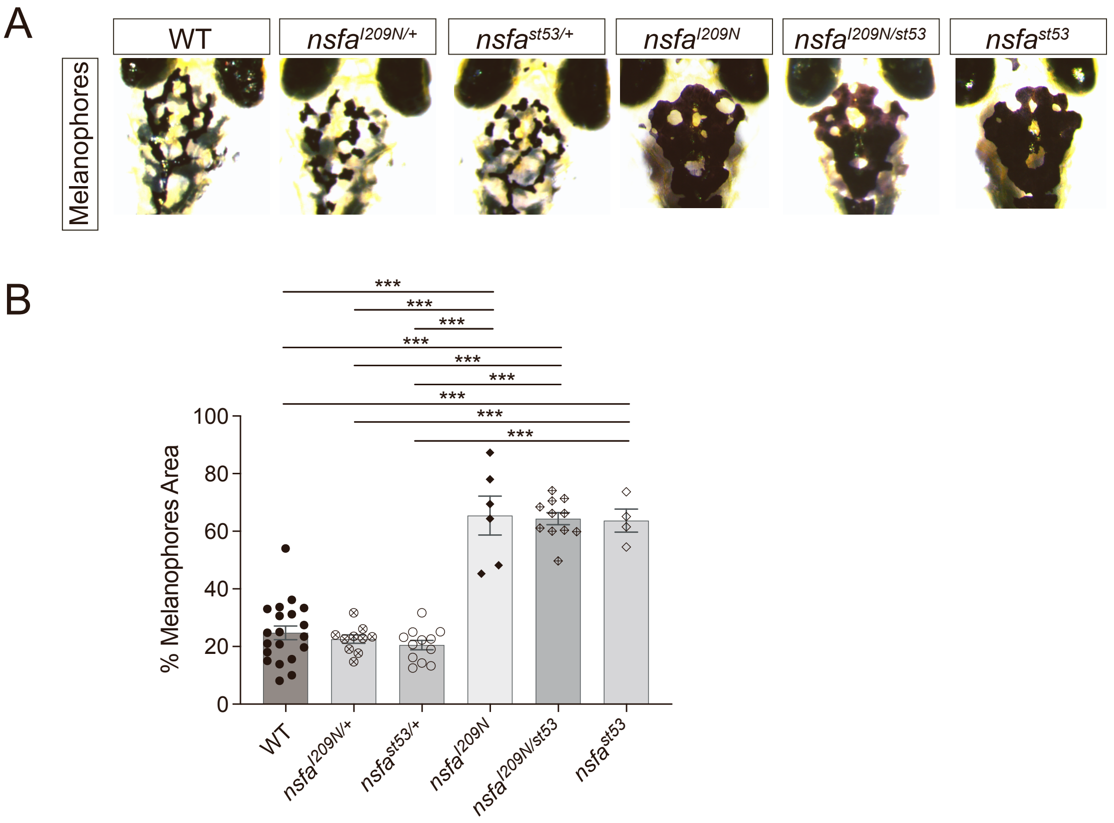

Supplemental Figure 1. Melanophores are expanded under bright conditions in both homozygous and compound nsfa mutants, Related to Figure 1.

(A) Top-down views of the darkly pigmented melanophores in homozygous and heterozygous WT larvae versus nsfaI209N homozygous mutants, nsfaI209N/st53 compound heterozygotes, and nsfast53 homozygous mutants.

(B) Quantification of the extent of melanophore expansion in a selected ROI from the melanophores located above the midbrain/hindbrain area. Quantification data are shown as mean ± SEM; P values are determined by a One-way ANOVA with Benjamini-Hochberg correction. *p<0.05, **p<0.01, ***p<0.001. For all experiments, n > 4 fish per genotype. All images and data are representative of 2 independent experiments.

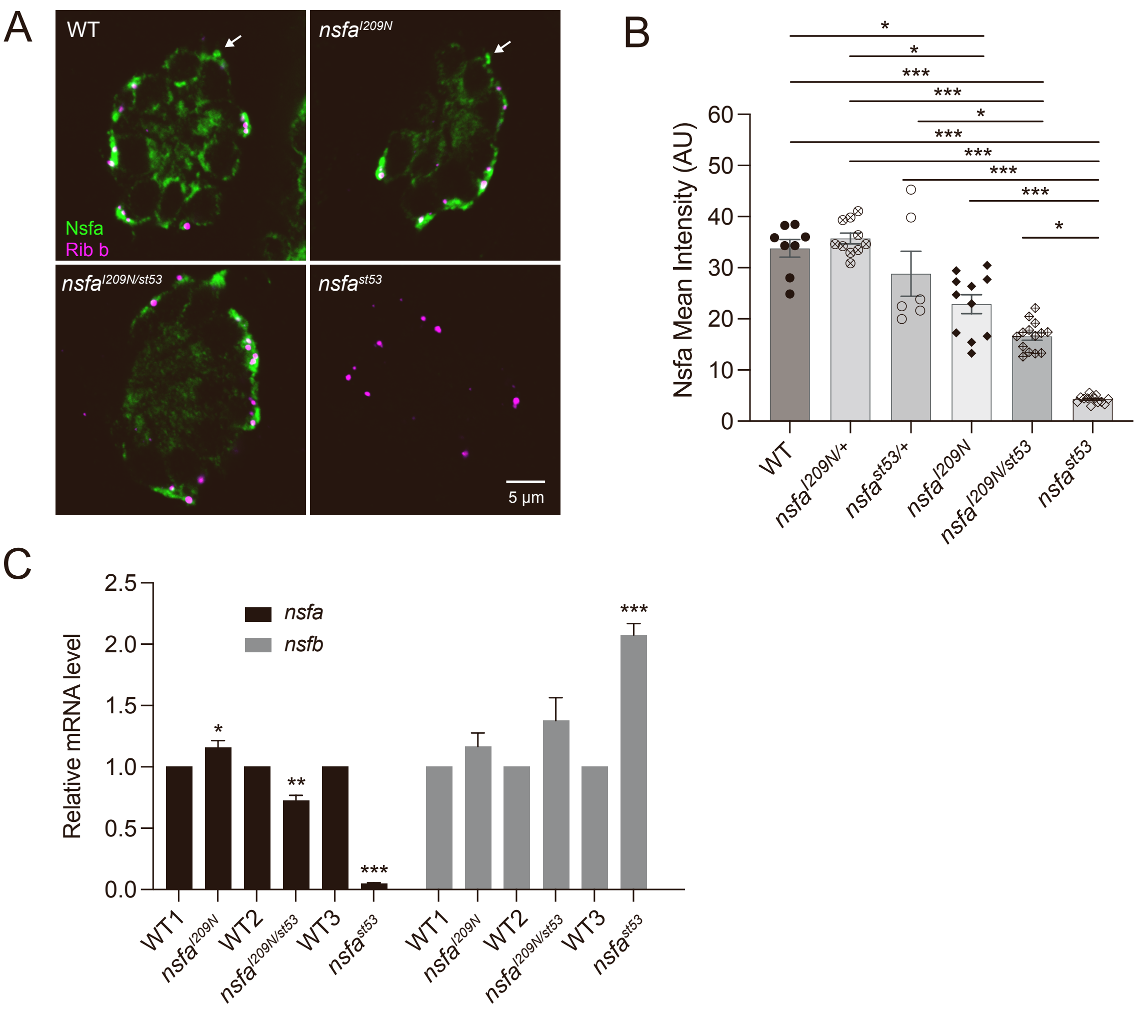

Supplemental Figure 2. Expression of Nsfa protein in lateral line hair cells and nsfa transcripts in whole fish, Related to Figure 1.

(A) Top-down views of Nsfa immunolabeling in neuromast hair cells. Single optical slices of the L1 neuromast are shown (5 dpf). Ribeye b (magenta) antibody labeling is used as a marker for presynaptic ribbons of hair cells. Nsfa (green) signals are seen in both hair cells and afferent synaptic terminals (white arrows).

(B) Quantification of the intensity of fluorescence of Nsfa in hair cells using a ROI of the immunolabeling present above the nucleus of individual cells. Quantification data are shown as mean ± SEM; P values are determined by a One-way ANOVA with Benjamini-Hochberg correction. *p<0.05, **p<0.01, ***p<0.001.

(C) Quantitative PCR of nsfa and nsfb transcripts in WT siblings, nsfaI209N, nsfaI209N/st53, and nsfast53 larvae. Reactions were performed in triplicate. The gene expression level in wild-type larvae was normalized to 1. Quantification data are shown as mean ± SEM; P values are determined by Student's t-tests for data in panel C. *p<0.05, **p<0.01, ***p<0.001. Scale bar = 5 µm.
